# Combining Lifestyle With Metformin to Reduce Dementia Risk: Updates From MET-FINGER Trial

**DOI:** 10.1093/geroni/igaf122.306

**Published:** 2025-12-31

**Authors:** Dinithi Perera, Mariagnese Barbera, Alina Solomon, Jenni Lehtisalo, Francesca Mangialasche, Lefkos Middleton, Tiia Ngandu, Miia Kivipelto

**Affiliations:** FINGERS Brain Health Institute, Stockholm, Stockholms Lan, Sweden; University of Eastern Finland, Kuopio, Pohjois-Savo, Finland; Karolinska Institutet, Solna, Stockholms Lan, Sweden; Finnish Institute for Health and Welfare, Helsinki, Uusimaa, Finland; Division of Clinical Geriatrics, Stockholm, Stockholms Lan, Sweden; Imperial College London, London, England, United Kingdom; Finnish Institute for Health and Wefare, Helsinki, Uusimaa, Finland; Karolinska Institutet, Solna, Stockholms Lan, Sweden

## Abstract

MET-FINGER is the first randomized controlled trial combining healthy lifestyle changes with a putative disease-modifying drug (metformin) for the prevention of cognitive impairment, in an APOEε4-enriched at-risk population of older adults. The potential benefits of metformin repurposing are based on the link between Type-II Diabetes (T2D) and Alzheimer’s Disease (AD), and emerging evidence of independent neuro-protective effects. Findings will provide crucial information for innovative precision-prevention strategies aimed at cognitive impairment and dementia risk-reduction. 600 participants are randomly allocated to the FINGER 2.0 intervention (lifestyle for all + metformin, where appropriate; active arm), or regular health advice (control arm). Participants in the FINGER 2.0 group and at increased risk of T2D, are further randomised to 2000mg/day metformin, 1000mg/day metformin, or placebo (double-blind). The multimodal lifestyle intervention is an optimised version of the FINGER model. Metformin is administered as Glucophage®XR/SR 500, (Merck Healthcare KGaA, Darmstadt, Germany; 500mg oral tablets). Primary outcome is change in global cognition (combined z-score of a neuropsychological test battery). Secondary outcomes are changes in individual cognitive domains; functional ability; and risk factors for AD/dementia. The feasibility, potential lifestyle-metformin interaction, and disease-modifying effects of the lifestyle-metformin combination are exploratory outcomes. 489 eligible participants have been identified; 365 have been randomised. The main challenges experienced are linked to balancing APOEε4-carriers enrichment and the eligibility rate for metformin treatment (e.g., APOEε4-carriers were less likely to fulfil some eligibility criteria for metformin). Lessons learned, pertaining to trial design, set-up, recruitment, and delivery, for future combination-therapy studies, will be presented.

